# A Prepubertal Mice Model to Study the Growth Pattern of Early Ovarian Follicles

**DOI:** 10.3390/ijms22105130

**Published:** 2021-05-12

**Authors:** Yingjun Chen, Qinghua Liu, Ruiyan Liu, Chan Yang, Xiaodong Wang, Zaohong Ran, Shanshan Zhou, Xiang Li, Changjiu He

**Affiliations:** 1 College of Animal Science and Technology, Huazhong Agricultural University, Wuhan 430070, China; cyjmail.hzau.edu.cn@webmail.hzau.edu.cn (Y.C.); 2017302110041@webmail.hzau.edu.cn (Q.L.); Ruiyan-Liu@webmail.hzau.edu.cn (R.L.); YangChan@webmail.hzau.edu.cn (C.Y.); 2015302200616@webmail.hzau.edu.cn (X.W.); RanZaohong@webmail.hzau.edu.cn (Z.R.); shanshanzhou@webmail.hzau.edu.cn (S.Z.); 2 Key Laboratory of Agricultural Animal Genetics, Breeding and Reproduction of Ministry of Education, Huazhong Agricultural University, Wuhan 430070, China; 3 National Center for International Research on Animal Genetics, Breeding and Reproduction, Huazhong Agricultural University, Wuhan 430070, China

**Keywords:** folliculogenesis, preantral follicle, oogenesis, ovary, puberty

## Abstract

Early folliculogenesis begins with the activation of the follicle and ends with the formation of the follicular antrum, which takes up most of the time of folliculogenesis. In this long process, follicles complete a series of developmental events, including but not limited to granulosa cell (GC) proliferation, theca folliculi formation, and antrum formation. However, the logical or temporal sequence of these events is not entirely clear. This study demonstrated in a mouse model that completion of early folliculogenesis required a minimum of two weeks. The oocyte reached its largest size in the Type 4–5 stage, which was therefore considered as the optimum period for studying oogenesis. Postnatal days (PD) 10–12 were regarded as the crucial stage of theca folliculi formation, as *Lhcgr* sharply increased during this stage. PD13–15 was the rapid growth period of early follicles, which was characterized by rapid cell proliferation, the sudden emergence of the antrum, and increased *Fshr* expression. The ovarian morphology remained stable during PD15–21, but antrum follicles accumulated gradually. Atresia occurred at all stages, with the lowest rate in Type 3 follicles and no differences among early Type 4–6 follicles. The earliest vaginal opening was observed at PD24, almost immediately after the first growing follicular wave. Therefore, the period of PD22–23 could be considered as a suitable period for studying puberty initiation. This study objectively revealed the pattern of early folliculogenesis and provided time windows for the study of biological events in this process.

## 1. Introduction

The ovary in the prepubertal female is not a dormant organ but is characterized by continuous follicle recruitment and growth, which is known as early folliculogenesis or gonadotropin-independent folliculogenesis [1]. Early folliculogenesis starts from the activation of primordial follicles and ends with the formation of small antral follicles. In this process, the early follicles go through four stages, namely primary (Type 3), early secondary (Type 4), middle secondary (Type 5a), and late secondary (Type 5b) [2,3,4]. Pituitary gonadotropins can affect early folliculogenesis, but are not essential, because follicles can develop into the late secondary follicular stage despite the lack of follicle-stimulating hormone (FSH) and luteinizing hormone (LH) signaling [5,6]. After antrum formation, the destiny of follicles is mainly determined by gonadotropins. They either mature under the co-regulation of FSH and LH or get eliminated by atresia in the case of insufficient gonadotropins [7]. As a result, early folliculogenesis occurs round after round in the prepubertal ovary. However, as the pituitary gland is not yet fully mature, none of the follicles can ovulate, which leads to a futile exhaustion of the ovarian follicle reserve [8]. Mice are unique in that they can only complete one round of early folliculogenesis before puberty due to the short interval between birth and puberty initiation [9]. Early folliculogenesis is regulated mainly by ovarian signals. Experimental works have suggested that transforming growth factor-beta family cytokines (including inhibin/activin, follistatin, and growth and differentiation factor 9) [10], FSH [11], C-type natriuretic peptide [12], melatonin [13], and hippo signaling [14] in the ovary play important roles in the regulation of early folliculogenesis.

The duration of the female’s reproductive period depends on the activation rate of the follicle pool. Thus, the amount of activated primordial follicles is tightly regulated, as abnormalities in primordial follicle activation can lead to premature ovarian failure (POF) and female infertility [15,16]. Studies have demonstrated that early-growing follicles can produce feedback signals to control the scale of activated primordial follicles. For example, anti-Müllerian hormone (AMH) made by early-growing follicles inhibits the activation of primordial follicles, and the absence of AMH leads to a faster depletion of primordial follicles and causes POF [17,18]. The FSH receptor (*Fshr*) is not expressed in primordial follicles but has been detected in GCs in the early-growing follicles. *Fshr*-null follicles do not develop beyond the preantral stage and thus disturb the process of follicle activation, which causes POF in one-year-old mice [5,19].

The above examples indicate that early-growing follicles play a key role in maintaining the female reproductive period. Nevertheless, research has mainly focused on the brief period of follicular activation rather than early folliculogenesis. This is partially due to the fact that follicular activation connects tightly to reproductive aging, and the morphological changes involved in it are relatively simple, which reduces the difficulty of designing research protocols [15,16]. By contrast, early folliculogenesis is a long and complicated process involving multiple stages, namely Type 3, Type 4, Type 5a, Type 5b, and Type 6. Furthermore, many biological events such as zona pellucida formation, oocyte enlargement, follicular theca formation, follicular antrum formation, and GC proliferationand differentiation occur in early folliculogenesis [2,20]. Therefore, without an accurate understanding of the developmental pattern of early follicles, it is difficult to formulate reasonable experimental designs for specific biological events, let alone study the entire process of early folliculogenesis.

The present study aims to sketch out a detailed map of early folliculogenesis and trace the “time window” for the study of biological events involved in it. To accomplish this, the first wave of activated follicles in newborn mice was used to explore the morphological pattern of early folliculogenesis, because it can provide a clean background and ideally eliminate the interruptions of follicles in other developmental stages. In brief, ovaries were collected daily during PD5–21 and subjected to histological analysis. The growth curves of the follicles and oocytes were recorded carefully, and the expression profiles of follicle-growth-related genes and puberty initiation were explored.

## 2. Results

### 2.1. Histological Analysis of the Dynamics of Early Folliculogenesis

**Figure 1:**
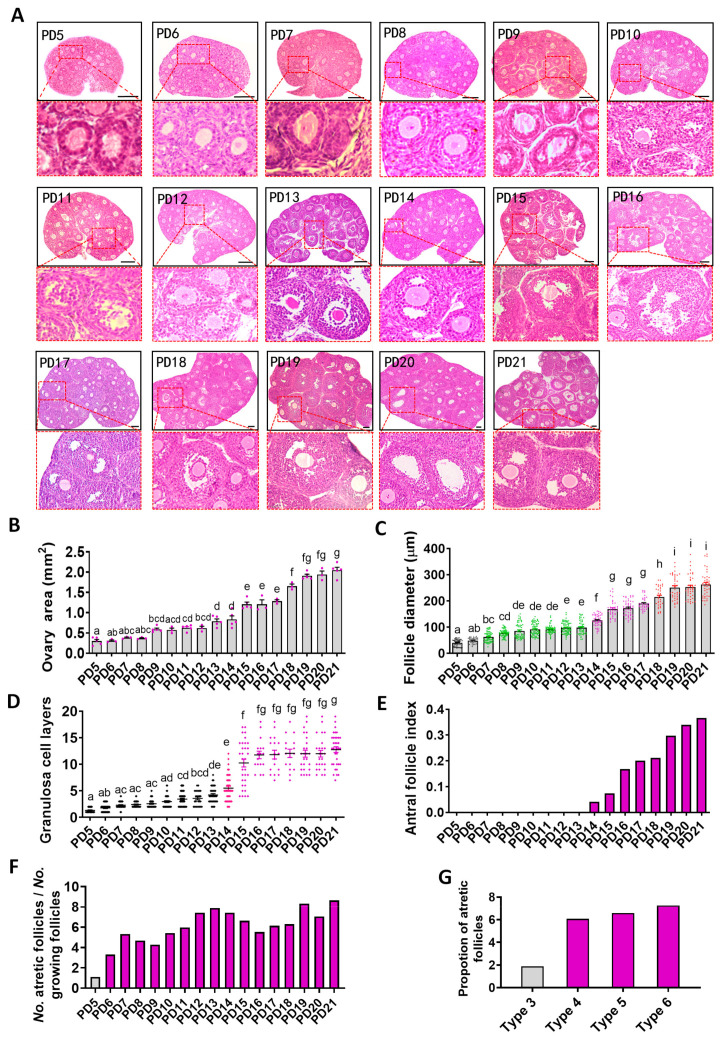
The development mode of follicles during prepubertal stage.(**a**) The representative HE staining of ovary from PD5 to 21. The scale bar = 100 μm. (**b**) Maximum cross-sectional area of ovaries (data are expressed as mean ± SEM). Three to five ovaries from 3–5 mice in each group were used for sectioning. Three to five sections in each group were used for these statistics. Statistical significance was determined using one-way ANOVA followed by Tukey post hoc test. (**c**) Follicle diameter (data are expressed as mean ± SEM). Thirty to forty-five sections from 3–5 mice in each group were used for these statistics. Statistical significance was determined using one-way ANOVA followed by Tukey post hoc test. (**d**) The number of GC layers (data are expressed as mean ± SEM). Thirty to forty-five sections from 3–5 mice in each group were used for these statistics. Statistical significance was determined using one-way ANOVA followed by Tukey post hoc test. (**e**) Antral follicle index. Thirty to forty-five sections from 3–5 mice on each day were counted. (**f**) Percentage of atretic follicles at different days of age. Thirty to forty-five sections from 3–5 mice on each day were counted. (**g**) Percentage of follicular atresia at different developmental stages. The number of follicles used for these statistics: *n* = 844 (Type 3), 986 (Type 4), 2308 (Type 5), 207 (Type 6). The difference is significant or not depends on whether one or more identical superscript letters (a–i) between groups exist (*p* > 0.05) or not (*p* < 0.05).

Ovaries were collected during PD5–21 and subjected to hematoxylin and eosin (HE) staining; the representative figures of each day are listed in Figure 1a. The maximum cross-sectional area was recorded as the ovarian area, and the data demonstrated that the ovaries underwent a period of slow development during PD5–12 (PD5: 0.30 ± 0.042 versus PD12: 0.62 ± 0.046 mm^2^, *p* < 0.05), after which two rapid developmental stages were observed on PD13–15 (PD13: 0.79 ± 0.056 versus PD15: 1.21 ± 0.054 mm^2^, *p* < 0.01) and PD17–19 (PD17: 1.29 ± 0.036 versus PD19: 1.91 ± 0.038 mm^2^, *p* < 0.01) (Figure 1b). Then, the follicle diameter, the number of layers of GCs, and the antral follicle index were measured to investigate the development pattern of early follicles. The pattern of change in follicle diameter was very similar to that of the ovary. It underwent a period of slow development during PD5–13 (PD5: 40.27 ± 1.94 versus PD13: 98.07 ± 3.83 μm, *p* < 0.01). Two rapid development stages were observed during PD13–15 (PD13: 98.1 ± 3.83 versus PD15: 168.5 ± 5.22 μm, *p* < 0.01) and during PD16–19 (PD16: 172.5 ± 4.04 versus PD19: 250.5 ± 7.20 μm, *p* < 0.01) (Figure 1c). The number of GC layers underwent a period of slow development during PD5–13 (PD5: 1.32 ± 0.09 versus PD13: 4.22 ± 0.25, *p* < 0.01), which was followed by rapid development during PD13–15 (PD13: 4.22 ± 0.25 versus PD15: 10.25 ± 0.75, *p* < 0.01). During PD15–21, the number of cell layers did not obviously change (Figure 1d). The process of follicular antrum formation was recorded; the antrum was visible on day 14 and then increased gradually during days 15 to 21 (Figure 1e). The proportion of atretic follicles was also calculated. We observed a rapid increase in atretic follicles on PD5 to 7, followed by a fluctuation of 4 to 8 percent (Figure 1f). Further analysis showed that atresia occurred at all follicular stages, with the lowest rate in Type 3 follicles and no difference among Type 4–6 follicles (Figure 1g).

### 2.2. Expression Patterns of Genes Related to Folliculogenesis

**Figure 2:**
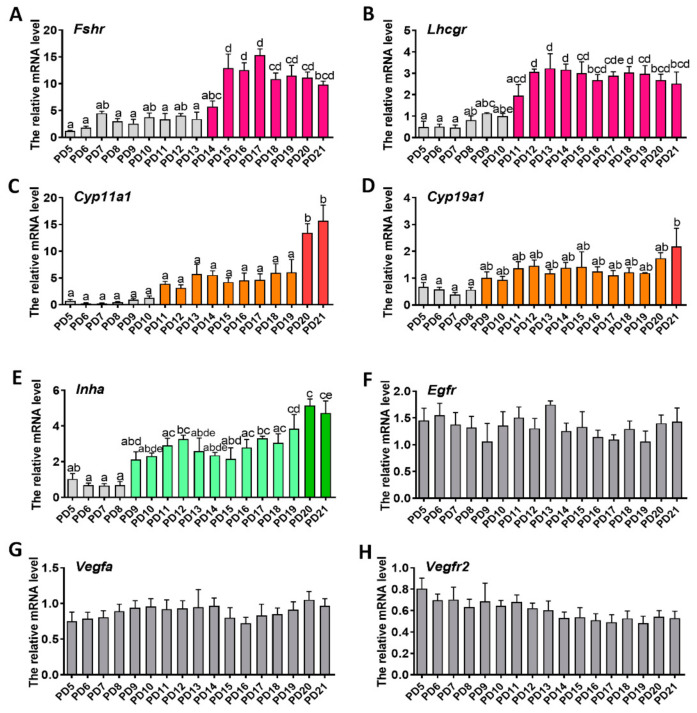
The expression patterns of genes associated with folliculogenesis during early folliculogenesis: (**a**) follicle-stimulating hormone receptor (*Fshr*); (**b**) luteinizing hormone/ choriogonadotropin receptor (*Lhcgr*); (**c**) cytochrome p450 family 11 subfamily-A member 1 (*Cyp11α1*); (**d**) cytochrome p450 family 19 subfamily-A member 1 (*Cyp19α1*); (**e**) inhibin-a subunit (*Inhα*); (**f**) epidermal growth factor receptor (*Egfr*); (**g**) vascular endothelial growth factor a (*Vegfα*); (**h**) vascular endothelial growth factor receptor 2 (*Vegfr2*). Three samples in each group were used for gene quantification. Normalization was performed using the housekeeping gene *Actb* as control. Data are expressed as mean ± SEM. Statistical significance was determined using one-way ANOVA followed by Tukey post hoc test. The difference is significant or not depends on whether one or more identical superscript letters (a–e) between groups exist (*p* > 0.05) or not (*p* < 0.05).

The expression patterns of genes associated with follicle development (*Fshr*, *Lhcgr*, *Cyp11α1*, *Cyp19α1*, *Inhα, Egfr*) were examined. Compared with the expression level on PD 5, *Fshr* was upregulated slowly during PD6–13 and increased sharply during PD13–15, after which the expression level remained unchanged (Figure 2a). By contrast, *Lhcgr* was upregulated slowly during PD6–10, and increased sharply during PD10–12; thereafter, the expression of *Lhcgr* remained consistent (Figure 2b). The expression of *Cyp11α1* kept consistent during PD5–8 and increased slowly during PD8–19, then increased sharply (Figure 2c). Similarly, the expression of *Cyp19α1* showed no significant change during PD5–8 and increased gradually during PD8–19, then increased quickly from PD19 to 21 (Figure 2d). The expression pattern of *Inhα* was similar to that of *Cyp19α1*: it kept consistent during PD5–8 and increased gradually from PD8 to 21 (Figure 2e). The expression of *Egfr* showed no significant variation during early folliculogenesis (Figure 2f). Similarly, no changes of expression levels for either *Vegfα* or *Vegfr2* mRNA were observed (Figure 2g,h).

### 2.3. Expression Patterns of Genes Related to Proliferation

**Figure 3:**
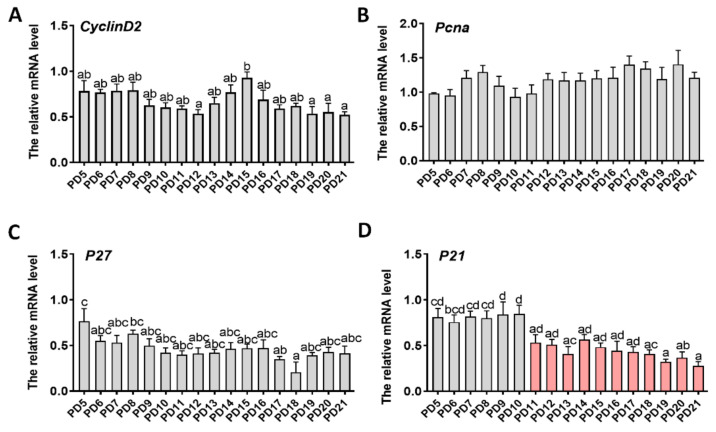
The expression patterns of genes associated with proliferation during early folliculogenesis: (**a**) G1/S-specific cyclin-D2 (*CyclinD2*); (**b**) proliferating cell nuclear antigen (*PCNA*); (**c**) cyclin-dependent kinase inhibitor 1 (*P27*); (**d**) cyclin-dependent kinase inhibitor 1B (*P21*). Three samples in each group were used for gene quantification. Normalization was performed using the housekeeping gene *Actb* as control. Data were expressed as mean ± SEM. Statistical significance was determined using one-way ANOVA followed by Tukey post hoc test. The difference is significant or not depends on whether one or more identical superscript letters (a–d) between groups exist (*p* > 0.05) or not (*p* < 0.05).

The expression patterns of genes associated with cell proliferation were also examined. *CyclinD2* was upregulated slightly during PD12–15 and down-regulated in later stages (Figure 3a). By contrast, both *PCNA* and *P27* showed no significant changes during early folliculogenesis (Figure 3b,c). The expression of *P21* remained consistent during PD5–10 and then decreased gradually from PD10 to 21 (Figure 3d).

### 2.4. Relationship between Oogenesis and Early Folliculogenesis

**Figure 4:**
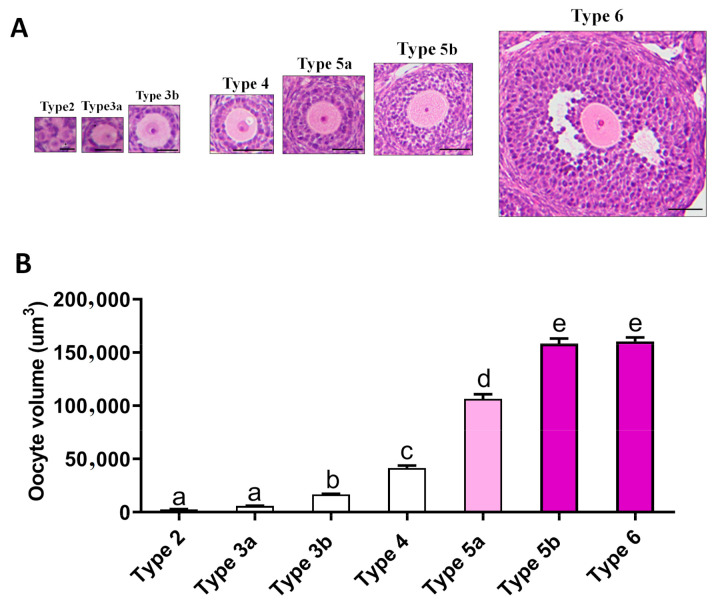
The relationship between oogenesis and folliculogenesis: (**a**) the selection criteria of the follicles, the scale bar = 20 μm (type 2–3b follicles), 50 μm (type 4–6 follicles); (**b**) the development pattern of the oocytes. Twenty oocytes in each phase were counted. Data are expressed as mean ± SEM. Statistical significance was determined using one-way ANOVA followed by Tukey post hoc test. The different superscript letters (a–e) represent a significant difference (*p* < 0.05).

It is widely believed that follicle development is necessary for oogenesis. It was particularly interesting for us to determine the growth pattern of oocytes during follicle development. It was observed that oocytes within a Type 2–3b follicle enlarged gradually (Type 2: 2740 ± 162.8 versus Type 3b: 16,789 ± 450.3 μm^3^, *p* < 0.01); those within a Type 4–5 follicle enlarged sharply, and the largest oocytes were observed within Type 5b follicles (Type 4: 41,506 ± 2202 versus Type 5b: 158,482 ± 4696 μm^3^, *p* < 0.01) (Figure 4). Subsequently, oocyte volume did not change. These data suggested that oogenesis is not synchronized with follicle development.

### 2.5. Identification of the Day-Age of Follicles Entering the Gonadotropin-Dependent Phase

**Figure 5:**
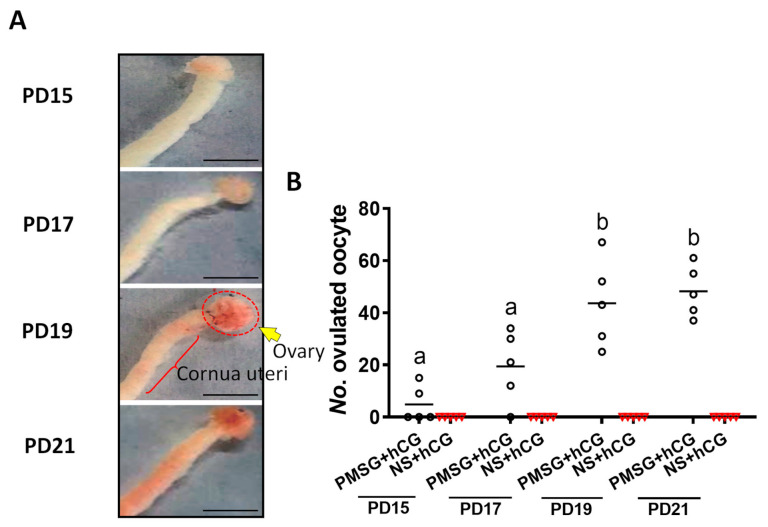
Identification of the day-age of follicles entering the gonadotropin-dependent phase: (**a**) representative reproductive organ pictures of each time point after gonadotropin injection, the scale bar = 5 mm; (**b**) number of ovulated oocytes. Five mice were subjected to superovulation in each group. Data are expressed as mean ± SEM. Statistical significance was determined using one-way ANOVA followed by Tukey post hoc test. The different superscript letters (a–b) represent a significant difference (*p* < 0.05).

Superovulation was conducted to determine the time when follicles responded to gonadotropic hormones by observing the changes in the size and congestion of the reproductive organs. The response of the reproductive organs to gonadotropin increased gradually during PD15–21 (Figure 5a). Ovulation was observed after the gonadotropin stimulation on PD15. The number of ovulated oocytes increased on PD17 (PD15: 4.8 ± 3.09 versus PD17: 19.4 ± 6.16, *p* > 0.05) and then reached its largest value during PD19–21 (PD19: 43.6 ± 7.49 versus PD21: 48.2 ± 4.41, *p* > 0.05). As we expected, hCG cannot independently induce ovulation without PMSG pretreatment (Figure 5b).

### 2.6. Identification of the Day-Age of Puberty Initiation

**Figure 6:**
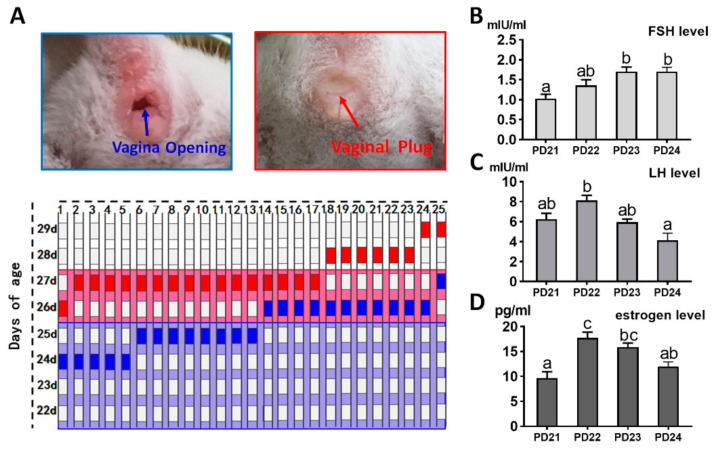
The day-age of puberty initiation and hormone variation pattern: (**a**) the day-age of vagina opening and first mating. Blue grid: vagina opening, red grid: first-mating. Twenty-five mice were used for vaginal examination and mating; (**b**–**d**) variation curves of FSH, LH, and estrogen. Six serum samples in each group were used for hormone assay. Data are expressed as mean ± SEM. Statistical significance was determined using one-way ANOVA followed by Tukey post hoc test. The difference is significant or not depends on whether one or more identical superscript letters (a–c) between groups exist (*p* > 0.05) or not (*p* < 0.05).

The day of vaginal opening and first mating were detected to confirm the competition time of puberty initiation. The average day of vaginal opening was 25.3 ± 0.17 day, and the average day of first mating age was 27.4 ± 0.14 day (Figure 6a). Compared with PD21, FSH levels on PD23 and 24 were significantly increased (PD21: 1.03 ± 0.106 versus PD23: 1.70 ± 0.115 mIU/mL, *p* < 0.05; PD21: 1.03 ± 0.106 versus PD24: 1.70 ± 0.108 mIU/mL, *p* < 0.05) (Figure 6b). By contrast, LH and estradiol-17β levels were at their highest levels on PD22 and then decreased gradually (Figure 6c,d).

## 3. Discussion

Newborn mouse ovaries are densely packed with germ cell cysts. The germ cell cysts are transformed into primordial follicles, which process is also known as the establishment of the follicular pool, during PD3–5 [21]. Thereafter, a certain proportion of primordial follicles are activated to form the first follicle growth wave. After a long process, most form a small follicular antrum, which is regarded as an indicator of the end of early folliculogenesis [22]. The first batch of activated follicles originating from the newborn ovary is a perfect model for studying early folliculogenesis because it can provide a clean background and ideally eliminate the interruption of follicles in other developmental stages. Previous research has used less frequent sampling. For example, Peters used a 1 week sample interval and compared morphological differences between PD 1, 7, 14, and 21 [23]. Infrequent sampling cannot trace the entire process of folliculogenesis, as it would miss some crucial biological events. In this study, we collected the ovaries from PD5 to 21 and subjected them to histomorphological analysis, which is currently the most detailed description of the model of early folliculogenesis in mice.

This study showed that early folliculogenesis consisted of three phases: slow growth, rapid growth, and slow growth (Figure 1). During the first slow growth phase, comprising the interval of PD5–13, the activated follicles grew slowly to the Type 5b stage. During PD13–15, follicles underwent a rapid growth and formed a small antrum, which marked the acquisition of the ability to respond to gonadotropins. Therefore, the interval of PD13–15 is a crucial phase in transformation of the early follicle into a gonadotropin-dependent follicle. However, the exact signal and how it is activated during PD13–15 is still unknown. During the interval of PD15 to 21, the growth of the early follicles slowed down again and the ovaries changed rarely in morphology, but antral follicles accumulated gradually. It is believed that the first batch of small antral follicles play an important role in regulating puberty initiation [24]; therefore, the period of PD15–21 may also be the preparation phase for puberty initiation. Atresia occurred at all follicular stages [25]. Earlier studies have demonstrated that the majority of follicles undergo atresia at the small antral follicular stage [26,27], because small antrum follicles often fail to have adequate access to FSH, which is essential for survival [28]. This study once again confirmed that atresia occurred at all stages of early follicle development. However, atresia of secondary follicles was not less than that of small antral follicles (Figure 1f,g). The above findings further the understanding of follicular atresia.

*Fshr* and *Lhcgr* act as antennas on the follicle, where they are responsible for receiving gonadotropin signals from the pituitary gland [29,30,31]. In the current study, we found that both *Fshr* and *Lhcgr* were gradually upregulated at the early stage of folliculogenesis; soon afterwards, they underwent a period of rapid increase and then reached a plateau. The rapid increase of *Lhcgr* emerged on days 10 to 12. *Lhcgr* is mainly expressed in the theca folliculi of the early follicle [32,33]. Thus, we presume that the period of PD10 to 12 is the crucial stage for theca folliculi formation. Notably, the expression of *Fshr* experienced at least two periods, namely the slow increase period before follicular antrum formation, and the rapid increase period during follicular antrum formation. Therefore, the increase of *Fshr* may be an important prerequisite for the formation of the follicular antrum. This finding also helps us to understand why follicles can still develop into the late secondary follicles after *Fshr* deletion, but the follicular antrum cannot be further formed [5,19]. In other words, it inspired us to use *Fshr* as a breakthrough point to study the formation of the follicular antrum. For example, signals or genes that induce a sharp upregulation of *Fshr* may be involved in the regulation of antrum formation. The time point of follicle entrance to the gonadotropin-dependent phase was investigated. It was observed that oocytes started to ovulate on PD15 when *Fshr* expression reached the highest level. Superovulation experiments also indicated that the completion of early folliculogenesis required a minimum of two weeks, which is much longer than the two day time of gonadotropin-dependent folliculogenesis [31]. *Cyp11α1*, *Cyp19α1*, *Inhα*, *Egfr*, *Vegfα*, and *Vegfr2* are the downstream genes of FSH-FSHR signaling. Among them, *Cyp11α1* and *Cyp19α1* control the synthesis of sex steroids [34], and *Inhα* influences gonadotropin secretion [35]. These three genes were all upregulated and then reached their highest levels on PD21, just before puberty initiation. Thus, these genes may provide molecular markers of puberty initiation.

Deletion of *CyclinD2* impairs GC proliferation and prevents follicle development [36]. In the current study, *CyclinD2* increased slightly during PD12–15, which coincided with the rapid growth of the follicle. Initiation of the cell cycle involves the inhibition of endogenous Cdk inhibitors (e.g., *P21* and *P27*), which inhibit the activity of cyclin/Cdk complexes [37]. In the current study, the expression of *P27* gradually decreased after day 10, while that of *P21* did not change significantly. We also found that GCs proliferated in a restricted manner and formed an antrum when the cells went beyond 10 layers. Notably, the layers of GCs did not increase further after the forming of the follicular antrum. It seems that the layers (or cell amounts) of GCs may act as a timer within the follicle to drive GC differentiation and antrum formation. This would be similar to the emergence of the blastocyst cavity, wherein the blastomere cannot initiate differentiation and secrete fluids to form the blastocyst cavity until its cell number increases to a certain value at morula stage [38,39]. However, the mechanism by which the follicle senses the amount of GCs is unknown.

Our findings suggest that oogenesis is not synchronized with folliculogenesis. The oocyte underwent a rapid expansion during the very early stage and reached its largest size in the Type 4–5 stage. During the subsequent process, the oocyte reached a steady state even though follicle growth was just beginning. Previous studies have also confirmed that oocytes do not grow during gonadotropin-dependent folliculogenesis. Instead, the nuclear maturation capacity of oocytes is acquired during early folliculogenesis [40,41,42]. The reason why folliculogenesis is slower than oogenesis is also currently unclear. We presume that this development model is an important strategy of reproductive regulation that evolved during the evolution. In this model, the oocyte is the core of female reproduction, while follicle acts as a “liaison“ between the oocyte and the nerve center: the gonadal hormones released by the follicle allow the nerve center to perceive the mature state of the oocyte; in turn, the nerve center promotes estrogen secretion and produces a preovulatory LH peak, thereby accurately controlling the mating and ovulation time of females and ensuring that the ovulated oocytes have sufficient fertilization opportunities.

This study shows that puberty occurs mainly on PD24–26, much earlier than the day 34 observed by Peters [23]. Therefore, we conclude that PD22–23 may be a “time window” for studying puberty initiation. In particular, most of the mice exhibited mating activity within 2 days of PD22–23, suggesting that mice can mate naturally even when they weigh much less than adults. This finding can be used as a reference for the design of related experiments. During puberty initiation, the basal level of LH was much higher than that of FSH. Moreover, changes in LH and estrogen concentrations were very similar. Since the main role of LH is to induce the theca folliculi to synthesize the precursor of estrogen, we speculated that the ability of estrogen synthesis in prepubertal mice may also be determined by LH. Therefore, LH may play a more important role during puberty initiation than FSH. This idea has also been put forward by researchers in earlier years [43]. Notably, the release of FSH and LH is pulsatile in a day; hence, the level of these hormones is time-dependent [44]. Therefore, a shorter sampling interval may be used to depict the change in hormones accurately.

## 4. Materials and Methods

### 4.1. Mice Handing

Kunming mice were purchased from the Experimental Animal Center of Huazhong Agricultural University (Wuhan, China). Mice were housed under suitable temperature (22–26 °C) with constant 12 h light-dark cycles and allowed access to food and water ad libitum. All animal experiments were carried out with the approval of the Animal Ethics Committee of Huazhong Agricultural University. The approval number is HZAUMO–2018–060. Euthanasia was performed using gradual increase in CO_2_ concentration or cervical dislocation.

### 4.2. Histomorphological Analysis

Ovaries were collected on PD5, 6, 7, 8, 9, 10, 11, 12, 13, 14, 15, 16, 17, 18, 19, 20, and 21 (*n* = 3–5 per group). Then the samples were fixed in 4% paraformaldehyde at room temperature for 48 h (G1101, Servicebio technology, Wuhan, China). After fixation, the ovaries were washed in phosphate buffer and embedded in paraffin. Complete ovaries were serial sectioned (5 μm-thick paraffin sections), and every fifth section was chosen for HE staining. The images were taken by a microscope (Olympus, Tokyo, Japan) connected to a computer. Ovary area and follicle diameter were measured by Image J software (National Institutes of Health, Bethesda, MD, USA). In brief, the area of all sections derived from the same ovary was determined, and the maximum value was selected as the area of ovary; the diameter of the growing follicle was measured; and the number of GC layers was counted. Since early folliculogenesis is a continuous process, we measured the diameter and GC layers of all growing follicles in each section in order to accurately show the growth curve of early follicles, and then arranged the follicle diameter and cell layer number in descending order. Finally, the top 20% of values were used to generate Figure 1. The antral follicle index was calculated as the number of antral follicles divided by the number of growing follicles. The area of oocytes originating from follicles at different developmental phases was measured. Follicles were classified according to Pedersen’s criterion [4].

### 4.3. Gene Expression Assay with Real-Time qPCR

The ovaries were collected on PD5, 6, 7, 8, 9, 10, 11, 12, 13, 14, 15, 16, 17, 18, 19, 20, and 21. Three samples at each time point were used for gene quantification. The samples were ground in liquid nitrogen. Total RNA was extracted using Trizol reagent (Invitrogen Inc., Carlsbad, CA, USA). Thereafter, the expression of genes associated with folliculogenesis (*Fshr*, *Lhcgr*, *Cyp11α1*, *Cyp19α1*, *Inhα*, *Egfr*, *Vegfα*, *Vegfr2*) and proliferation (*CylinD2*, *PCNA*, *P21*, *P27*) was determined as follows:

RNA was reverse transcription using a PrimeScript^TM^ RT reagent kit with genome DNA Eraser (RR047A, Takara Bio Inc., Tokyo, Japan). The following was the reaction procedure of qPCR. qPCR analysis was performed using a QuantiFast SYBR Green PCR Kit on a Bio-Rad CFX Manager Machine (Bio-Rad, Hercules, CA, USA). The qPCR reaction contained SYBR Green (10 μL), forward and reverse primers (500 nM for each), template cDNA (8 μL), and added ddH_2_O to make a total volume of 20 μL. The reaction procedure was as follows: predegeneration 95 °C for 10 min; 35 cycles of denaturation at 95 °C for 10 s and annealing/extension at 60 °C for 15 s; and melting curve from 65–95 °C, increasing in increments of 0.5 °C every 5 s. The relative RNA levels were normalized to those of housekeeping gene *Actb*. The relative mRNA expression was calculated by the 2^−^^△△ct^ method. Primer sequences are listed in Table 1.

### 4.4. Superovulation

Mice were injected with 5 IU PMSG (B191009, Ningbo Hormone Products Co., Ltd. Zhejiang, China) to stimulate follicle growth on PD15, 17, 19, and 21 (*n* = 5 per group). Forty-four hours after PMSG injection (the control group mice were not injected with PMSG), hCG at a dose of 5 IU (S180801, Ningbo Hormone Products Co., Ltd. Zhejiang, China) was injected to trigger ovulation. Fourteen hours after hCG injection, mice were sacrificed, and the reproductive organs were collected for visual observation. MII-stage oocytes were collected and counted by puncturing the oviduct.

### 4.5. Puberty Initiation Determination

After PD21, the state of the vagina was determined every morning, and opening of the vagina was considered as a sign of puberty. The pubertal mice were then collected and allowed to cohabitate with a male that night. Mating was confirmed by the presence of a vaginal plug.

### 4.6. FSH, LH, and Estradiol-17β Level Analyses by Radioimmunoassay

The serum concentrations of FSH, LH, and estradiol-17β were measured on PD21, 22, 23, and 24 (*n* = 6 per group). In brief, blood was collected from the caudal vein. After clotting, the serum was obtained by centrifugation at 3000 rpm for 10 min and stored at −20 ℃. The levels of FSH, LH, and estradiol-17β were measured by radioimmunoassay. These hormone levels were measured by the Beijing North Institute of Biological Technology (Being, China). The FSH, LH, and estradiol-17β detection kits were all purchased from the Jiancheng Bioengineering Institute (Nanjing, China).

### 4.7. Statistics Analysis

GraphPad Prism 8.0 software (GraphPad Software Inc., San Diego, CA, USA) was used for data statistical analysis. Data were presented as the mean ± standard error of mean (SEM). The statistical analysis was performed by one-way analysis of variance (ANOVA) followed by the Tukey post hoc test and chi-square test. *p*-value < 0.05 was considered statistically significant; *p*-value < 0.01 was considered highly statistically significant.

## 5. Conclusions

The current study demonstrated that: completion of early folliculogenesis required a minimum of two weeks; the oocyte reached its largest size in Type 4–5 stage, after which they stop enlarging; PD10–12 could be the crucial stage of theca folliculi growth; PD13–15 was a period of rapid growth of early follicles, during which the follicular antrum began to form; atresia occurred at all follicular stages, with a higher atretic percent in Type 4–6 follicles; puberty onset almost immediately the end of the first growing follicular wave. In short, we drew a detailed atlas of early folliculogenesis and provided time windows for studying oocyte growth, theca folliculi formation, follicular antrum formation, follicular atresia, and puberty initiation, thereby laying the foundation for further investigation of the regulatory mechanisms underlying early folliculogenesis.

## Figures and Tables

**Table 1:** The primers for qPCR.

Genes	Primer Sequence (5’–3’)	Product Size (bp)	Tm (°C)
*Actb*	Forward: CCAGCCTTCCTTCTTGGGTAT	93	60
	Reverse: AGGTCTTTACGGATGTCAACG		
*Lhcgr*	Forward: CTGAGGAGATTTGGTTGCTGTA	234	60
	Reverse: ATTTGGGTGGACTTTTTTGGGG		
*Cyp11α1*	Forward: GGGCAGTTTGGAGTCAGTTTAC	186	60
	Reverse: TTTAGGACGATTCGGTCTTTCTT		
*Fshr*	Forward: GCAGATGTGTTCTCCAACCTACC	172	60
	Reverse: GGAGAGACTGGATCTTGTGAAAGG		
*Egfr*	Forward: AAGGCACAAGTAACAGGCTCA	114	60
	Reverse: CCAAGTTCCCAAGGACCACT		
*Cyp19α* *1*	Forward: GACACATCATGCTGGACACC	179	60
	Reverse: CAAGTCCTTGACGGATCGTT		
*Vegfα*	Forward: GAGAAGACAGGGTGGTGGAAG	193	60
	Reverse: GAAGGGAAGATGAGGAAGGGT		
*Vegfr2*	Forward: CCTGCCTACCTCACCTGTTTC	205	60
	Reverse: CCACTGTCTGTCTGGCTGTC		
*Inhα*	Forward: CTTTCCCTCTGCTGACCCA	184	60
	Reverse: AAAGCCGCAGGAGACCAA		
*CyclinD2*	Forward: GCTATGGAGCTGCTGTGCT	263	60
	Reverse: CCAAGAAACGGTCCAGGTAA		
*PCNA*	Forward: ACCTGCAGAGCATGGACTCG	83	60
	Reverse: GCAGCGGTATGTGTCGAAGC		
*P21*	Forward: CCTGGTGATGTCCGACCTG	130	60
	Reverse: CCATGAGCGCATCGCAATC		
*P27*	Forward: TCAAACGTGAGAGTGTCTAACG	238	60
	Reverse: CCGGGCCGAAGAGATTTCTG		

## Data Availability

The data presented in this study are available in the article and on request from the corresponding author.
